# Isolation of Alginate-Degrading Bacteria from the Human Gut Microbiota and Discovery of *Bacteroides xylanisolvens* AY11-1 as a Novel Anti-Colitis Probiotic Bacterium

**DOI:** 10.3390/nu15061352

**Published:** 2023-03-10

**Authors:** Tianyu Fu, Yamin Wang, Mingfeng Ma, Wei Dai, Lin Pan, Qingsen Shang, Guangli Yu

**Affiliations:** 1 Key Laboratory of Marine Drugs of Ministry of Education, Shandong Key Laboratory of Glycoscience and Glycotechnology, School of Medicine and Pharmacy, Ocean University of China, Qingdao 266003, China; 2 Laboratory for Marine Drugs and Bioproducts, Qingdao National Laboratory for Marine Science and Technology, Qingdao 266003, China; 3 Qingdao Marine Biomedical Research Institute, Qingdao 266071, China

**Keywords:** *Bacteroides xylanisolvens*, gut microbiota, alginate, ulcerative colitis, dextran sulfate sodium, probiotic, marine polysaccharides, degradation, inflammatory bowel disease, fermentation

## Abstract

Alginate has been documented to prevent the development and progression of ulcerative colitis by modulating the gut microbiota. However, the bacterium that may mediate the anti-colitis effect of alginate has not been fully characterized. We hypothesized that alginate-degrading bacteria might play a role here since these bacteria could utilize alginate as a carbon source. To test this hypothesis, we isolated 296 strains of alginate-degrading bacteria from the human gut. *Bacteroides xylanisolvens* AY11-1 was observed to have the best capability for alginate degradation. The degradation and fermentation of alginate by *B. xylanisolvens* AY11-1 produced significant amounts of oligosaccharides and short-chain fatty acids. Further studies indicated that *B. xylanisolvens* AY11-1 could alleviate body weight loss and contraction of colon length, reduce the incidences of bleeding and attenuate mucosal damage in dextran sulfate sodium (DSS)-fed mice. Mechanistically, *B. xylanisolvens* AY11-1 improved gut dysbiosis and promoted the growth of probiotic bacteria, including *Blautia* spp. And *Prevotellaceae UCG-001*, in diseased mice. Additionally, *B. xylanisolvens* AY11-1 showed no oral toxicity and was well-tolerated in male and female mice. Altogether, we illustrate for the first time an anti-colitis effect of the alginate-degrading bacterium *B. xylanisolvens* AY11-1. Our study paves the way for the development of *B. xylanisolvens* AY11-1 as a next-generation probiotic bacterium.

## 1. Introduction

Alginate is a functional marine polysaccharide that is widely used in the food and pharmaceutical industries [1]. As a dietary fiber, alginate is not absorbed after oral intake [2]. Therefore, when reaching the distal colon, it is degraded, fermented and utilized by the intestinal microbiota [2]. The fermentation and utilization of alginate by specific microbes in the gut could change the structure and composition of the intestinal microbiome and confer benefits to the host by producing beneficial short chain fatty acids (SCFAs) [3].

Inflammatory bowel disease (IBD), including ulcerative colitis and Crohn’s disease, is emerging worldwide at an alarming rate [4]. Accumulating evidence indicates that changes in the gut microbiota play a pivotal role in the pathogenesis of ulcerative colitis [4]. For example, some nonpathogenic *Escherichia coli* strains stimulate the release of proinflammatory cytokines and initiate the inflammatory cascade in the colon [4]. However, other probiotic bacteria, including *Faecalibacterium prausnitzii*, *Parabacteroides distasonis* and *Lactobacillus casei*, protect the intestinal mucosa and epithelial cells from chemically induced inflammatory responses in the colon [4,5]. It is worth mentioning that nutrition and daily diet exert a strong influence on the development and progression of inflammatory bowel disease [6]. Recently, the dietary intake of alginate has been documented to prevent the development of ulcerative colitis by modulating the gut microbiota [7]. Mechanistically, alginate increases the intestinal abundance of a probiotic bacterium, *Bifidobacterium animalis*, and promotes the biosynthesis of hyodeoxycholic acid in the gut [7]. The anti-colitis effect of alginate is evidenced to be mediated by specific gut microbes as it is poorly absorbed in the intestine [2,3,7]. However, although *Bifidobacterium animalis* has been proposed as a functional bacterium to mediate the beneficial effect of alginate on the colon, whether other bacteria exist that could play a similar role in the gut has yet to be determined.

Our previous results indicate that alginate is readily degraded and fermented by specific anaerobes in the human gut [2,3]. We hypothesized that the alginate-degrading bacteria may mediate the anti-colitis effect of alginate since these bacteria can utilize this polysaccharide to promote their growth. To test this hypothesis, we isolated 296 strains of alginate-degrading bacteria from the human gut and found that *B. xylanisolvens* AY11-1 has the best capability for the degradation of alginate. Interestingly, *B. xylanisolvens* AY11-1 was also observed to have a potent anti-colitis effect in dextran sulfate sodium (DSS)-fed mice. Our present study illustrates for the first time an anti-colitis effect of the alginate-degrading bacterium *B. xylanisolvens* AY11-1 and paves the way for the development of *B. xylanisolvens* AY11-1 as a next-generation probiotic bacterium.

## 2. Materials and Methods

### 2.1. Chemicals and Reagents

Alginate and its derivatives, including polymannuronate (PM) and polyguluronate (PG), were prepared as previously described [3]. Tryptone, peptone, yeast extract and Tween 80 were obtained from Sigma (Shanghai, China). Dextran sulfate sodium (DSS) was obtained from MP Biomedicals (Solon, OH, USA). Hemin, agar and L-cysteine hydrochloride were obtained from Sangon Biotech (Shanghai, China) Co., Ltd. (Shanghai, China). All other chemicals of analytical grade were purchased from Sinopharm Chemical Reagent Co., Ltd. (Shanghai, China) unless otherwise specified.

### 2.2. Isolation of Alginate-Degrading Bacteria from Human Gut Microbiota

The fresh fecal samples from 30 healthy volunteers were collected as previously described [3,8]. All individuals provided a signed consent before sample collection. The human experiments for the fecal sample collection were approved and supported by the Ethical Committee of Ocean University of China, School of Medicine and Pharmacy (Permission No. OUC-2020-1008-01) as previously described [3,8]. The VI medium that contained alginate at a concentration of 8 g/L was utilized to isolate the alginate-degrading bacteria from the human gut microbiota using the method described elsewhere [3,8,9]. Total carbohydrate analysis using the well-established phenol–sulfuric acid method was applied to quantify the utilization of the polysaccharides [3,8,9]. Phylogenetic tree analysis of the alginate-degrading bacteria was performed using the Molecular Evolutionary Genetics Analysis (MEGA) software (version 7.0.26). Structural analyses of the degradation products using Fourier transform mass spectrometry (MS) and short-chain fatty acids (SCFAs) analyses of the fermentation products using high-performance liquid chromatography (HPLC) were conducted as previously described [3,8,9].

### 2.3. Animals and Treatment

All the specific pathogen-free (SPF) animals were obtained from Beijing Vital River Laboratory Animal Technology Co., Ltd. (Beijing, China) (Certificate No. SCXK (Jing) 2016-0011). The animal experiments were approved by the Ethical Committee of Ocean University of China, School of Medicine and Pharmacy (Permission No. OUC-2021-0301-03), and all the experiments were performed in compliance with the Guide for the Care and Use of Laboratory Animals (National Academies Press, 8th edition, 2011).

In the animal experiment 1, a total of 24 male 8-week-old C57BL/6J SPF mice were utilized to investigate the anti-colitis effect of *B. xylanisolvens* AY11-1. The 24 male mice were divided into 3 different groups: the normal control group (NC, *n* = 8), the model group (MD, *n* = 8) and the *B. xylanisolvens* AY11-1 treatment group (BX, *n* = 8). *B. xylanisolvens* AY11-1 was cultured in the VI medium for approximately 24 h before harvesting for the animal experiments. *B. xylanisolvens* AY11-1 was dissolved in an anaerobic phosphate-buffered saline (PBS). *B. xylanisolvens* AY11-1 was given at a dosage of approximately 1.05 × 10^7^ colony forming units (CFUs)/day/mouse with gavage. Mice in the MD group and the BX group were given 2.0% (*w*/*v*) DSS in their daily drinking water for 6 consecutive days. All mice were humanely sacrificed on the 7th day. The colon and cecum tissues of the mice were harvested for further experiments. The stool morphology, bleeding occurrence and body weight of the mice were monitored every day. The symptom scores of the mice were calculated based on the stool morphology, bleeding occurrence and body weight change in the mice at the 7th day. The histopathological colon scores of the mice were calculated based on the hematoxylin and eosin (H&E) staining results. The Alcian blue staining and the H&E staining of the mouse colon tissues were performed using the method previously described [5,10].

In the animal experiment 2, a total of 20 male and 20 female 8-week-old C57BL/6J SPF mice were employed to investigate the safety of *B. xylanisolvens* AY11-1. The 40 mice were divided into 4 different groups: the male normal control group (NCM, *n* = 10), the male *B. xylanisolvens* AY11-1 treatment group (BXM, *n* = 10), the female normal control group (NCF, *n* = 10) and the female *B. xylanisolvens* AY11-1 treatment group (BXF, *n* = 10). As detailed above, *B. xylanisolvens* AY11-1 was cultured in the VI medium for approximately 24 h before harvesting for the animal experiments. *B. xylanisolvens* AY11-1 was dissolved in an anaerobic PBS. *B. xylanisolvens* AY11-1 was given at a dosage of approximately 1.00 × 10^8^ CFUs/day/mouse with gavage. All mice were humanely sacrificed on the 28th day. The blood, heart, lung, liver, spleen, kidney, colon and cecum tissues of the mice were collected for further experiments. The blood cells of the mice were analyzed using the automatic blood cell analyzer (RT-7600Vet) from Rayto Life and Analytical Sciences Co., Ltd. (Shenzhen, China). The H&E staining was performed using the liver, spleen, kidney and colon tissues from the male and female mice as previously described [5,10].

### 2.4. High-Throughput Sequencing and Bioinformatic Analyses

The metagenomic DNA of the intestinal microbiota from animal experiment 1 was extracted from the cecum samples using a Qiagen QIAamp DNA Stool Mini Kit (Hilden, Germany). The 16S V3-V4 hypervariable gene regions were specifically amplified using the universal primers 338F (ACTCCTACGGGAGGAGCAG) and 806R (GGACTACHVGGGTWTCTAAT) as documented previously [3,5]. The obtained amplicons were sequenced on an Illumina PE300 platform from Majorbio Bio-pharm Biotechnology Co., Ltd. (Shanghai, China). The bioinformatic analyses of the 16S sequencing data, including Linear discriminant analysis Effect Size (LEfSe), Venn diagram, non-metric multidimensional scaling (NMDS) score plot, principal components analysis (PCA) and heatmap analysis were all conducted using the Majorbio Cloud Platform (www.majorbio.com (accessed on 10 December 2022)). For the LEfSe analysis, only the taxa with an LDA score above 3.5 were listed.

For the characterization of the alginate-degrading bacteria, the 16S genes of the isolates were first amplified using the universal primers 27F (AGAGTTTGATCMTGGCTCAG) and 1492R (GGTTACCTTGTTACGACTT) and then sequenced using the Applied Biosystems 3730XL DNA Analyzer from Sangon Biotech (Shanghai) Co., Ltd. (Shanghai, China). The taxonomic assignments of the isolated bacteria were performed using the EzBioCloud Database as previously described [11,12].

### 2.5. Statistical Analyses

All the data were expressed as the mean ± standard error of mean (SEM). The statistical analyses were performed using the Student’s *t*-test and ANOVA with post-hoc Tukey’s tests (GraphPad Prism; GraphPad Software Inc., San Diego, CA, USA). The results were considered statistically significant at *p* < 0.05. * *p* < 0.05 versus NC group; ** *p* < 0.01 versus NC group; # *p* < 0.05 versus MD group; ## *p* < 0.01 versus MD group.

## 3. Results

### 3.1. B. xylanisolvens AY11-1 Is a Potent Alginate-Degrading Bacterium in the Human Gut

**Figure 1:**
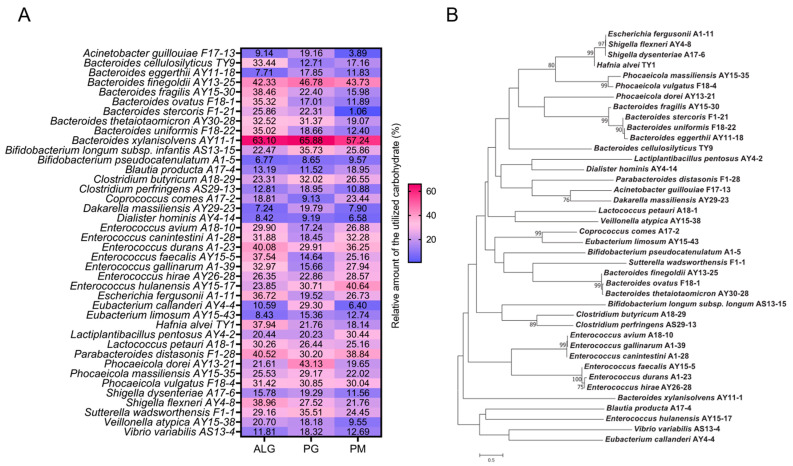
Isolation of alginate-degrading bacteria from the human gut microbiota. Phylogenetic tree analysis of the alginate-degrading bacteria (**A**). Heatmap analysis of the alginate-degrading capabilities of the isolated bacteria (**B**). The number indicates the relative amount of alginate that was utilized by the bacteria after 48 h of fermentation. Only one representative strain in each genus was used for the above analyses. Alginate (ALG), polyguluronate (PG), polymannuronate (PM).

Using the method previously developed by the authors [13], we isolated a total of 296 strains of alginate-degrading bacteria from the human gut (Table S1). These 296 strains belonged to 40 different species of bacteria (Figure 1, Table S1). *B. xylanisolvens* was the most frequently isolated species, and a total of 49 strains were obtained from the human gut (Table S1). Interestingly, *B. xylanisolvens* AY11-1, a representative strain from *B. xylanisolvens*, was also found to have the best capability for alginate degradation (Figure 1A). *B. xylanisolvens* AY11-1 could degrade and ferment approximately 60% of the alginate, polymannuronate (PM) and polyguluronate (PG) in 48 h (Figure 1A). *B. xylanisolvens* AY11-1 is not closely related to other *Bacteroides* spp., indicating that this bacterium may have evolved to specifically degrade and utilize alginate and its derivatives (Figure 1B).

Apart from *B. xylanisolvens* AY11-1, *Bacteroides finegoldii* AY13-25, *Enterococcus durans* A1-23 and *Parabacteroides distasonis* F1-28 were all identified as potent degraders of alginate (Figure 1A). *B. finegoldii* AY13-25, *E. durans* A1-23 and *P. distasonis* F1-28 could degrade and ferment approximately 30% to 50% of the alginate, PM and PG in 48 h (Figure 1A). *Acinetobacter guillouiae* F17-13, *Bifidobacterium pseudocatenulatum* A1-5 and *Dialister hominis* AY4-14 were identified as ineffective degraders of alginate as these bacteria could only use approximately less than 10% of the alginate in 48 h (Figure 1A).

**Figure 2:**
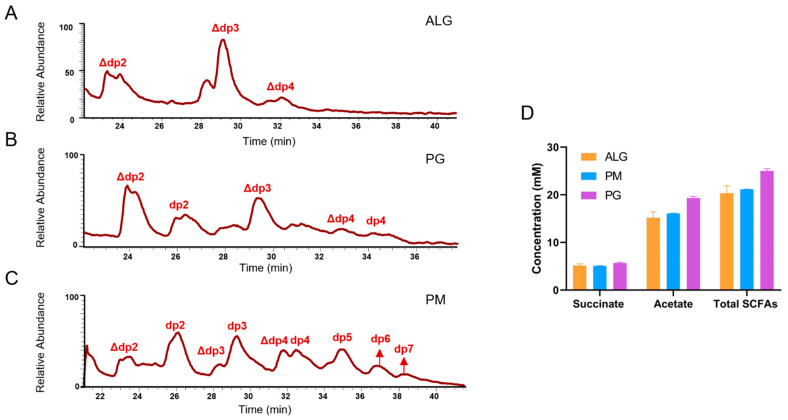
Degradation and fermentation of alginate and its derivatives by *B. xylanisolvens* AY11-1. Degradation products analysis (**A**–**C**). Fermentation products analysis (**D**). Alginate (ALG), polyguluronate (PG), polymannuronate (PM).

Since *B. xylanisolvens* AY11-1 was identified as the most efficient degrader of alginate, we further investigated its degradation products and fermentation products using mass spectrometry and high-performance liquid chromatography. Interestingly, the degradation of alginate by *B. xylanisolvens* AY11-1 produced significant amounts of unsaturated oligosaccharides with a degree of polymerization ranging from two to four (Figure 2A). However, degradation of PG by *B. xylanisolvens* AY11-1 produced significant amounts of both saturated and unsaturated oligosaccharides with a degree of polymerization ranging from two to four (Figure 2B). Degradation of PM by *B. xylanisolvens* AY11-1 produced significant amounts of both saturated and unsaturated oligosaccharides with a degree of polymerization ranging from two to seven (Figure 2C). The fermentation of alginate and its derivatives by *B. xylanisolvens* AY11-1 produced significant amounts of SCFAs, including acetate and succinate (Figure 2D). Altogether, our results indicate that *B. xylanisolvens* AY11-1 is a proficient degrader and fermenter of alginate and its derivatives in the human gut.

### 3.2. B. xylanisolvens AY11-1 Ameliorated Ulcerative Colitis in DSS-Fed Mice

Alginate has been documented to prevent the development and progression of ulcerative colitis by modulating gut microbiota [14,15,16]. However, the bacterium that may mediate the anti-colitis effect of alginate has not been fully characterized. Recently, *Bifidobacterium animalis* has been proposed as a functional bacterium to mediate the therapeutic effect of alginate on the colon [7]. However, whether other bacteria exist that could play a similar role in the gut has yet to be determined. We hypothesized that the alginate-degrading bacteria might play a role here since these bacteria could ferment alginate and produce beneficial SCFAs.

**Figure 3:**
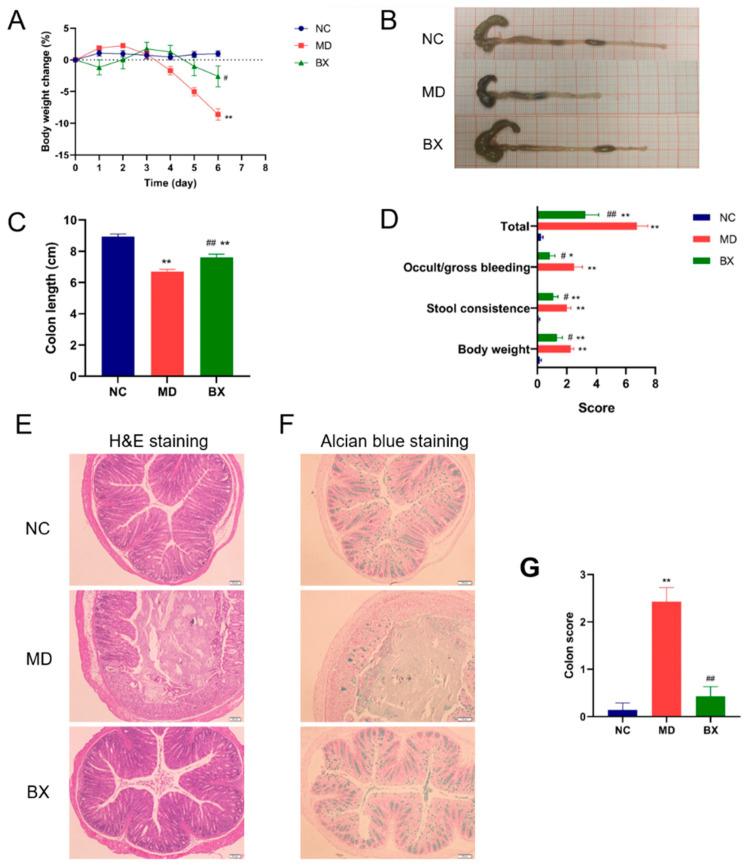
*B. xylanisolvens* AY11-1 ameliorated ulcerative colitis in mice. Body weight change in the mice (**A**). Morphological changes in the colon (**B**). Colon length (**C**). Symptom score analysis of the severity of ulcerative colitis (**D**). H&E staining (**E**) and Alcian blue staining (**F**) of the colon tissues. Histopathological score analysis of the colon (**G**). * *p* < 0.05 versus NC group; ** *p* < 0.01 versus NC group; # *p* < 0.05 versus MD group; ## *p* < 0.01 versus MD group.

To test this hypothesis, we next explored the anti-colitis effect of *B. xylanisolvens* AY11-1 using a DSS-induced ulcerative colitis model in C57BL/6J mice. Interestingly, we observed that oral administration of *B. xylanisolvens* AY11-1 improved ulcerative colitis in DSS-fed mice. Remarkably, *B. xylanisolvens* AY11-1 retarded the loss of body weight, reduced the shortening of the colon length, decreased the incidences of occult and gross bleeding and improved stool consistency in the DSS-fed mice (Figure 3A–D). The H&E staining and Alcian blue staining indicated that the oral intake of *B. xylanisolvens* AY11-1 attenuated the mucosal damage in the DSS-fed mice (Figure 3E–G). Altogether, we provide conclusive evidence for the first time of a therapeutic effect of the alginate-degrading bacterium *B. xylanisolvens* AY11-1 on DSS-induced ulcerative colitis.

### 3.3. B. xylanisolvens AY11-1 Modulated the Gut Microbiome by Increasing the Abundances of Blautia spp. and Prevotellaceae UCG-001

**Figure 4:**
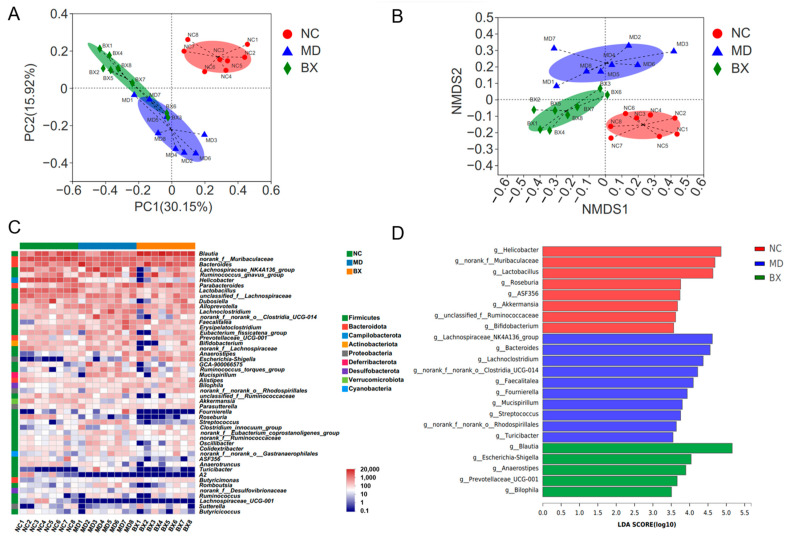
*B. xylanisolvens* AY11-1 altered the structure and modulated the composition of the gut microbiota. PCA score plot (**A**) and NMDS score plot analyses of the structure of the gut microbiota (**B**). Heatmap analysis of the gut microbiota at the genus level (**C**). LEfSe LDA score analysis of the gut microbiota (**D**). Only bacterial taxa with an LDA score of 3.5 are listed.

Preceding studies indicated a critical role of the gut microbiota in the development and treatment of ulcerative colitis [6,17,18]. In this regard, we next sought to explore the therapeutic mechanisms of *B. xylanisolvens* AY11-1 on inflammatory bowel disease from the viewpoint of intestinal microbiota. Venn diagram analysis suggested that a total of 13 different operational taxonomic units (OTUs) were exclusively identified in *B. xylanisolvens* AY11-1-treated mice (Figure S1). PCA and NMDS score plot analyses further confirmed that the oral intake of *B. xylanisolvens* AY11-1 could change the composition of the intestinal microbiome in diseased mice (Figure 4A,B).

To identify the functional gut microbes that were changed by the *B. xylanisolvens* AY11-1 treatment, we conducted the heatmap and LEfSe analyses (Figure 4C,D). Interestingly, the oral intake of *B. xylanisolvens* AY11-1 attenuated gut dysbiosis and increased the intestinal abundances of probiotic bacteria, including *Blautia* spp. and *Prevotellaceae UCG-001*, in diseased mice (Figure 4C,D). Collectively, these results suggest that *B. xylanisolvens* AY11-1 might have gut microbiota as its primary drug target during the treatment of ulcerative colitis.

### 3.4. B. xylanisolvens AY11-1 Showed No Oral Toxicity and Was Well-Tolerated in Both Male and Female Mice

**Figure 5:**
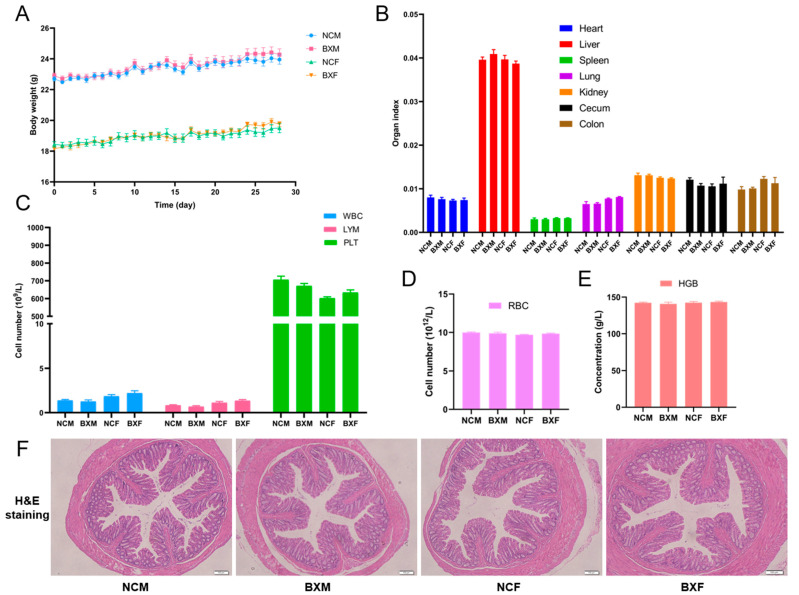
*B. xylanisolvens* AY11-1 showed no oral toxicity and was well-tolerated in both male and female mice. Changes in the body weight (**A**). Organ index (**B**). Cell counts of white blood cells (WBC), lymphocytes (LYM) and platelets (PLT) (**C**). Cell count of red blood cells (RBC) (**D**). Concentration of hemoglobin (HGB) (**E**). H&E staining of the colon tissues (**F**).

We next studied the safety profiles of *B. xylanisolvens* AY11-1 in healthy male and female mice. The healthy mice were given *B. xylanisolvens* AY11-1 orally at a dosage of approximately 10 times the dosage used for the colitis experiments. The mice were treated with *B. xylanisolvens* AY11-1 for four weeks, and we found that *B. xylanisolvens* AY11-1 was well-tolerated in both male and female mice (Figure 5). *B. xylanisolvens* AY11-1 showed no oral toxicity and did not affect the growth of the mice (Figure 5A,B). *B. xylanisolvens* AY11-1 treatment did not change the numbers of the blood cells, including white blood cells, lymphocytes, platelets and red blood cells (Figure 5C,D). Oral administration of *B. xylanisolvens* AY11-1 also had no effect on hemoglobin concentrations in the blood (Figure 5E). The H&E staining further confirmed that intake of *B. xylanisolvens* AY11-1 had no toxic effect on the major organs of mice, including the colon, liver, spleen and kidney (Figure 5F and Figure S2). Taken together, these results suggest that *B. xylanisolvens* AY11-1 was well-tolerated in both male and female mice. *B. xylanisolvens* AY11-1 could be used as a next-generation probiotic bacterium with good safety profiles.

## 4. Discussion

### 4.1. Future Perspectives and Potential Applications of B. xylanisolvens AY11-1

Preceding studies indicated that *Bacteroides ovatus*, *Bacteroides xylanisolvens* and *Bacteroides thetaiotaomicron* are major alginate-degrading bacteria in the human gut [2,3,8,19]. However, whether other bacteria exist that could also degrade and ferment alginate has not been investigated. Here, we identified 40 different species of intestinal bacteria that were able to degrade and ferment alginate. Our study extends previous research and provides new insights for understanding the bioactivity and metabolism of alginate in the gut.

The dietary intake of alginate is documented to prevent the development and progression of ulcerative colitis by modulating the gut microbiota [7,15]. We hypothesized that the alginate-degrading bacteria could play a role here, and interestingly, we found that *B. xylanisolvens* AY11-1, a proficient degrader of alginate, could alleviate ulcerative colitis in mice. Interestingly, *P. distasonis* F1-28, another alginate degrader from the human gut, was also found to have protective effects on DSS-induced ulcerative colitis [5]. Altogether, these results suggest that some of the beneficial effects of alginate on the colon may be mediated by specific microbes in the gut. Further investigations are needed to explore this possibility.

Previous studies indicated that specific strains from *B. xylanisolvens* could be used as next-generation probiotic bacteria with good safety profiles [20,21,22]. *B. xylanisolvens* DSM 23964 is a well-studied, next-generation probiotic (NGP) bacterium that has been isolated from human feces [20,22,23]. Recently, *B. xylanisolvens* DSM 23964 was authorized to be used as a food supplement in milk products by the European Commission [20,22,23]. In the present study, we illustrated that *B. xylanisolvens* AY11-1, a proficient degrader of alginate in the gut, could ameliorate ulcerative colitis and improve colonic mucosal damage in diseased mice. Additionally, we further demonstrated that *B. xylanisolvens* AY11-1 has a good safety profile and is well-tolerated in both male and female mice, even when dosed at 10 times the therapeutic concentration. Altogether, we demonstrate for the first time an anti-colitis effect of the alginate-degrading bacterium *B. xylanisolvens* AY11-1. Our present study paves the way for the development of *B. xylanisolvens* AY11-1 as a novel next-generation probiotic bacterium.

Our study provides a new framework for the discovery of novel probiotic bacteria from the human gut. Similar to fishing, the potential probiotic bacteria were hooked by the carbohydrate in the culture medium. This method could help to select specific carbohydrate-active bacteria from the gut. Using this strategy, we have successfully isolated 296 different alginate-degrading bacterial strains from the human gut and identified a novel bacterium with a potent anti-colitis effect. We anticipate that more probiotic bacteria could be discovered by applying this approach to other bioactive carbohydrates and functional molecules.

### 4.2. Limitations of the Current Study

Our study has some limitations. First, due to the COVID-19 pandemic, we were not able to get enough fecal samples to analyze and compare the compositional differences of the gut microbiota between healthy individuals and patients diagnosed with ulcerative colitis. In our present research, *B. xylanisolvens* was the most frequently isolated species, and a total of 49 different bacterial strains were obtained from the healthy human gut. It is highly possible that the protective bacterium *B. xylanisolvens* would be diminished in patients diagnosed with ulcerative colitis. However, more data are needed to verify this hypothesis.

Second, we have only isolated the alginate-degrading bacteria from healthy individuals. We do not know whether or not there would be any differences of the alginate-degrading bacteria in patients diagnosed with ulcerative colitis. More research is needed to fully address this issue. Third, the composition of the gut microbiota varies significantly among individuals. In the present study, we have only successfully isolated 296 alginate-degrading bacterial strains from 30 individuals. Undoubtedly, the sample size is not very big. In this regard, future studies should focus on expanding the sample size, and this would definitely help to obtain more alginate-degrading bacteria from the human gut. Additionally, new culture and isolation techniques should be created and employed to discover more carbohydrate-active bacteria from the human gut.

## 5. Conclusions

In conclusion, alginate and its derivatives could be degraded and fermented by approximately 40 different species of intestinal bacteria, including *B. xylanisolvens*, *B. finegoldii*, *P. distasonis* and *E. durans*. These alginate-degrading bacteria could possibly mediate the anti-colitis effect of alginate. Degradation of alginate, PM and PG by *B. xylanisolvens* AY11-1 produced significant amounts of oligosaccharides and beneficial short-chain fatty acids. Oral administration of *B. xylanisolvens* AY11-1 alleviated body weight loss and contraction of colon length, reduced incidences of bleeding and attenuated mucosal damage in dextran sulfate sodium (DSS)-fed mice. Mechanistically, *B. xylanisolvens* AY11-1 improved gut dysbiosis and promoted the growth of probiotic bacteria, including *Blautia* spp. and *Prevotellaceae UCG-001*, in diseased mice. Additionally, *B. xylanisolvens* AY11-1 showed no oral toxicity and was well-tolerated in male and female mice. Our results suggest that *B. xylanisolvens* AY11-1 is a good candidate for the development of next-generation probiotic bacteria.

## Data Availability

The data presented in this study are available on request from the corresponding authors.

## Supplementary Materials

The following supporting information can be downloaded at: www.mdpi.com/article/10.3390/nu15061352/s1, Table S1: Isolation of alginate-degrading bacteria from the human gut microbiota. Figure S1: Venn diagram analysis of the OTUs in different treatment groups. Figure S2: H&E staining of the liver, spleen and kidney tissues in different treatment groups.

## References

[1] Bi D., Yang X., Yao L., Hu Z., Li H., Xu X., Lu J. (2022). Potential food and nutraceutical applications of alginate: A Review. Mar. Drugs.

[2] Shang Q., Jiang H., Cai C., Hao J., Li G., Yu G. (2018). Gut microbiota fermentation of marine polysaccharides and its effects on intestinal ecology: An overview. Carbohydr. Polym..

[3] Fu T., Pan L., Shang Q., Yu G. (2021). Fermentation of alginate and its derivatives by different enterotypes of human gut microbiota: Towards personalized nutrition using enterotype-specific dietary fibers. Int. J. Biol. Macromol..

[4] Manichanh C., Borruel N., Casellas F., Guarner F. (2012). The gut microbiota in IBD. Nat. Rev. Gastroenterol. Hepatol..

[5] Ma M., Fu T., Wang Y., Zhang A., Gao P., Shang Q., Yu G. (2022). Polysaccharide from edible alga *Enteromorpha clathrata* improves ulcerative colitis in association with increased abundance of *Parabacteroides* spp. in the gut microbiota of dextran sulfate sodium-fed mice. Mar. Drugs.

[6] Mentella M.C., Scaldaferri F., Pizzoferrato M., Gasbarrini A., Miggiano G.A.D. (2020). Nutrition, IBD and gut microbiota: A review. Nutrients.

[7] Pi Y., Zhang X., Wu Y., Wang Z., Bai Y., Liu X., Han D., Zhao J., Tobin I., Zhao J. (2022). Alginate alleviates dextran sulfate sodium-induced colitis by promoting *Bifidobacterium animalis* and intestinal hyodeoxycholic acid synthesis in mice. Microbiol Spectr..

[8] Fu T., Zhou L., Fu Z., Zhang B., Li Q., Pan L., Zhou C., Zhao Q., Shang Q., Yu G. (2022). Enterotype-specific effect of human gut microbiota on the fermentation of marine algae oligosaccharides: A preliminary proof-of-concept in vitro study. Polymers.

[9] Pan L., Ai X., Fu T., Ren L., Shang Q., Li G., Yu G. (2021). In vitro fermentation of hyaluronan by human gut microbiota: Changes in microbiota community and potential degradation mechanism. Carbohydr. Polym..

[10] Pan L., Fu T., Cheng H., Mi J., Shang Q., Yu G. (2022). Polysaccharide from edible alga *Gloiopeltis furcata* attenuates intestinal mucosal damage by therapeutically remodeling the interactions between gut microbiota and mucin *O*-glycans. Carbohydr. Polym..

[11] Yoon S.H., Ha S.M., Kwon S., Lim J., Kim Y., Seo H., Chun J. (2017). Introducing EzBioCloud: A taxonomically united database of 16S rRNA gene sequences and whole-genome assemblies. Int. J. Syst. Evol. Microbiol..

[12] Park S.C., Won S. (2018). Evaluation of 16S rRNA Databases for Taxonomic Assignments Using Mock Community. Genom. Inform..

[13] Shang Q., Yin Y., Zhu L., Li G., Yu G., Wang X. (2016). Degradation of chondroitin sulfate by the gut microbiota of Chinese individuals. Int. J. Biol. Macromol..

[14] Mirshafiey A., Khodadadi A., Rehm B.H., Khorramizadeh M.R., Eslami M.B., Razavi A., Saadat F. (2005). Sodium alginate as a novel therapeutic option in experimental colitis. Scand. J. Immunol..

[15] Niu W., Chen Y., Wang L., Li J., Cui Z., Lv J., Yang F., Huo J., Zhang Z., Ju J. (2022). The combination of sodium alginate and chlorogenic acid enhances the therapeutic effect on ulcerative colitis by the regulation of inflammation and the intestinal flora. Food Funct..

[16] Razavi A., Khodadadi A., Eslami M.B., Eshraghi S., Mirshafiey A. (2008). Therapeutic effect of sodium alginate in experimental chronic ulcerative colitis. Iran. J. Allergy Asthma Immunol..

[17] Lavelle A., Sokol H. (2020). Gut microbiota-derived metabolites as key actors in inflammatory bowel disease. Nat. Rev. Gastroenterol. Hepatol..

[18] Schirmer M., Garner A., Vlamakis H., Xavier R.J. (2019). Microbial genes and pathways in inflammatory bowel disease. Nat. Rev. Microbiol..

[19] Li M., Li G., Shang Q., Chen X., Liu W., Pi X., Zhu L., Yin Y., Yu G., Wang X. (2016). In vitro fermentation of alginate and its derivatives by human gut microbiota. Anaerobe.

[20] Tan H., Zhai Q., Chen W. (2019). Investigations of *Bacteroides* spp. towards next-generation probiotics. Food Res. Int..

[21] O′Toole P.W., Marchesi J.R., Hill C. (2017). Next-generation probiotics: The spectrum from probiotics to live biotherapeutics. Nat. Microbiol..

[22] Brodmann T., Endo A., Gueimonde M., Vinderola G., Kneifel W., de Vos W.M., Salminen S., Gómez-Gallego C. (2017). Safety of novel microbes for human consumption: Practical examples of assessment in the European Union. Front. Microbiol..

[23] Wang C., Zhao J., Zhang H., Lee Y.K., Zhai Q., Chen W. (2021). Roles of intestinal *Bacteroides* in human health and diseases. Crit. Rev. Food Sci. Nutr..

