# Supplementary material for: Isolation of Alginate-Degrading Bacteria from the Human Gut Microbiota and Discovery of Bacteroides xylanisolvens AY11-1 as a Novel Anti-Colitis Probiotic Bacterium
Source: Nutrients. 2023 Mar 10;15(6):1352. doi: 10.3390/nu15061352 (PMC10053142; doi:10.3390/nu15061352)
Supplement: Supplementary file 1 [file nutrients-15-01352-s001.zip › nutrients-2263396-supplementary.pdf]

Table S1. Isolation of different alginate-degrading bacteria from the human gut microbiota.

No.	Alginate-degrading bacteria	Number of strains
1	*Acinetobacter guillouiae*	3
2	*Bacteroides finegoldii*	34
3	*Bacteroides ovatus*	7
4	*Bacteroides stercoris*	1
5	*Bacteroides thetaiotaomicron*	11
6	*Bacteroides uniformis*	3
7	*Bacteroides xylanisolvens*	49
8	*Escherichia fergusonii*	10
9	*Parabacteroides distasonis*	2
10	*Phocaeicola dorei*	7
11	*Phocaeicola massiliensis*	6
12	*Phocaeicola vulgatus*	9
13	*Shigella flexneri*	6
14	*Sutterella wadsworthensis*	2
15	*Hafnia alvei*	1
16	*Bacteroides cellulosilyticus*	1
17	*Bifidobacterium pseudocatenulatum*	8
18	*Blautia producta*	3
19	*Clostridium butyricum*	1
20	*Coprococcus comes*	2
21	*Enterococcus avium*	5
22	*Enterococcus canintestini*	6
23	*Enterococcus durans*	39
24	*Enterococcus faecalis*	7
25	*Enterococcus gallinarum*	1
26	*Lactococcus petauri*	2
27	*Shigella dysenteriae*	3
28	*Bifidobacterium longum subsp. infantis*	2
29	*Vibrio variabilis*	20
30	*Clostridium perfringens*	1
31	*Dakarella massiliensis*	3
32	*Lactiplantibacillus pentosus*	8
33	*Eubacterium callanderi*	2
34	*Dialister hominis*	1
35	*Bacteroides fragilis*	14
36	*Enterococcus hulanensis*	4
37	*Eubacterium limosum*	1
38	*Enterococcus hirae*	9
39	*Bacteroides eggerthii*	1
40	*Veillonella atypica*	1
Total	-	296

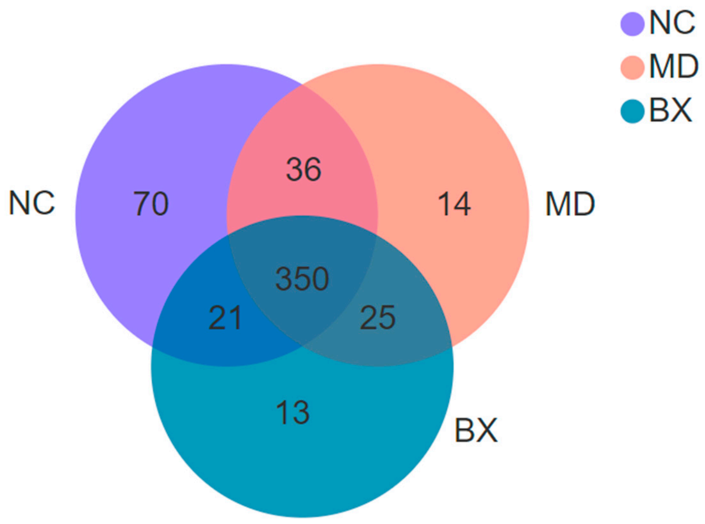

Figure S1. Venn diagram analysis of the OTUs in different treatment groups.

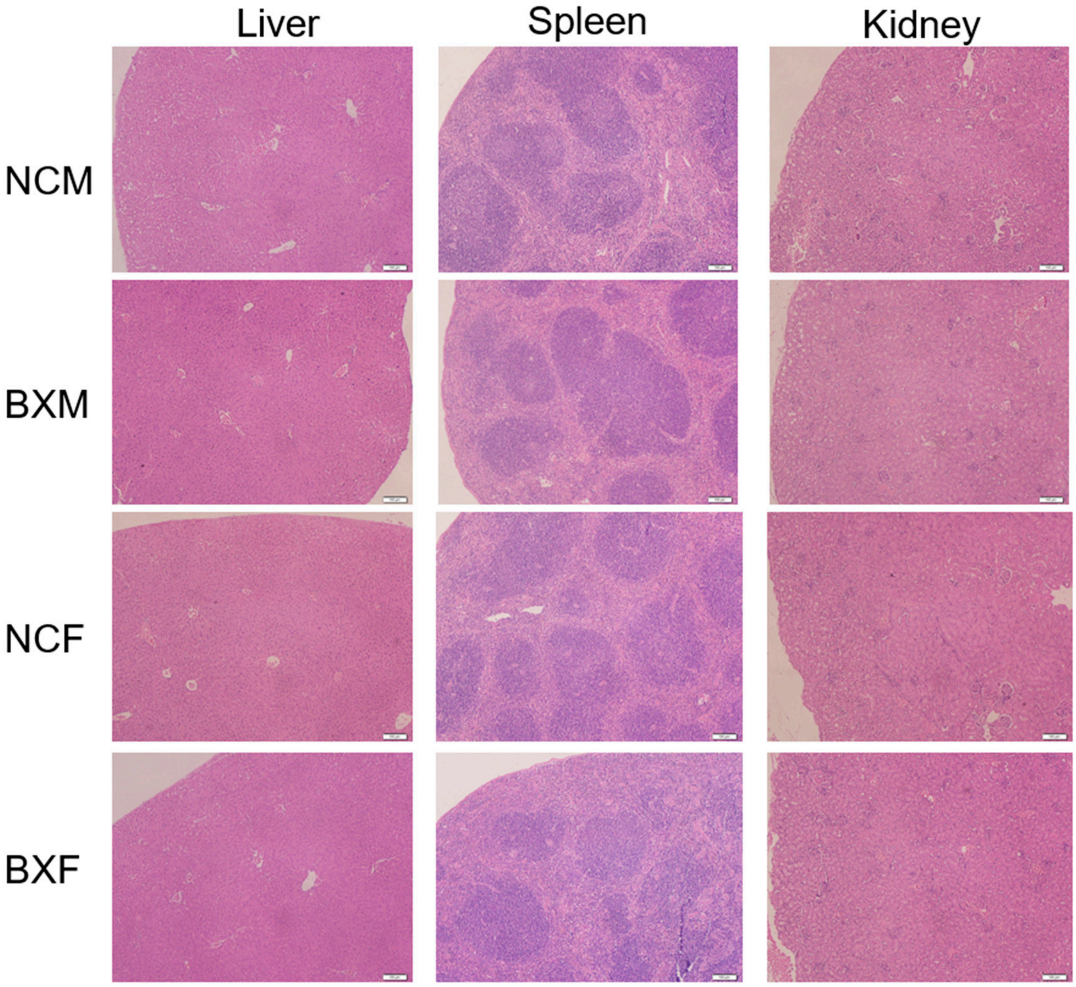

Figure S2. H&E staining of the liver, spleen and kidney tissues in different groups.
